# Circulating viral core and E1 antigen levels as supplemental markers for HCV Chronic hepatitis

**DOI:** 10.1186/1743-422X-3-67

**Published:** 2006-09-01

**Authors:** Mostafa K El Awady, Yasmine S El Abd, Hussein A Shoeb, Ashraf A Tabll, Alaa El Din MS Hosny, Reem M El Shenawy, Khaled Atef, Noha G Bader El Din, Mahmoud M Bahgat

**Affiliations:** 1 Department of Biomedical Technology, the National Research Center, Dokki, Egypt; 2 Department of Microbiology, Faculty of Pharmacy, Cairo University, Cairo, Egypt; 3 Department of Medicinal Chemistry, the National Research Center, Dokki, Giza, Egypt

## Abstract

The performance of polyclonal monospecific rabbit anti-sera raised against synthetic peptides derived from conserved HCV sequences of genotype 4 was evaluated for efficient detection of viral core and E1 antigens in circulating immune complexes (ICs) precipitated from 65 serum samples of HCV patients. The infection was established in those patients by the presence of HCV RNA in their sera. A novel enzyme-linked immunosorbent assay (ELISA) was developed for the detection of HCV core and E1 antigen in serum samples. Western blot analyses were used to demonstrate the presence of the core and E1 target antigen in serum samples. The mean OD readings of both core and E1 antigens were significantly higher (P < 0.05) among the viremic patients when compared to controls. Also a significant positive correlation (P < 0.05, r = 0.98) between the values of both core and E1 was recorded. Western blot analysis based on monospecific antibodies against core and E1 recognized the 38-kDa and 88 -kDa bands respectively in the sera of all infected patients. No specific reaction was observed with the sera from uninfected individuals. Interestingly the results of core and E1 antigen levels displayed no positive correlation with the HCV copy number as measured by bDNA. Liver enzymes (ALT and AST) showed a moderate positive correlation (r = 0.44 and 0.47 respectively) with the viral core antigens level. The same trend holds true for E1 (r = 0.43 and 0.64 for ALT and AST respectively). HCV load in infected patients revealed extremely poor correlation with serum ALT and AST levels (r = 0.022 and 0.002 respectively). In conclusion we present a new combination of serological tools correlating with liver enzyme levels that could be utilized as supplemental tests to viral load testing. Also, a sensitive and specific immunoassay was developed for the detection of HCV core and E1 in human serum. This test can be applied for laboratory diagnosis of HCV infection.

## Background

The genome of Hepatitis C Virus (HCV) consists of 5' and 3' untranslated regions that flank a single open reading frame (ORF) encoding structural and non-structural proteins [1] The structural proteins of HCV include the capsid (core) protein and two envelope glycoproteins (E1 and E2). Around 80% of infected individuals develop chronic infections, more than 2% of the globe is chronically infected and infection is the main etiological agent of chronic hepatitis, liver cirrhosis and hepatocellular carcinoma [2] Among the mechanisms the virus exerts to persist infection are, down regulating expression of its glycoproteins on cell surface, thus reducing the possibility of antibody recognition and destruction of infected cells, interference with the correct expression of major histocompatibility complexes (MHCs) on the cell surface or blocking antiviral immune responses such as the release of interferons (IFNs) and complement-mediated lysis [3,4], however, it is still not clear whether liver damage is directly caused by infection or results from host's immune reactions. Accumulation of mutations during viral replication results in a significant genetic heterogeneity and this may be attributed to lack of RNA-dependent RNA polymerase proofreading activity [1]. These mutations result in production of closely related, yet heterogeneous sequences so called quasispecies [[5-7] and [8]]. Such mutations within either the envelop or the core proteins may allow blocking of the viral infectivity or increase its aggression, respectively [9]. It is accepted that each quasispecies functions independently different from other mutants in terms of its response to neutralizing antibodies (escape mutants) and induction of liver damage. This was clearly demonstrated in chimpanzee where antibodies against HVR1 sequence of a quasispecies could neutralize its infection but did not function with other quasispecies [10] and also in patients who develop chronic HCV infection where the immune system is not entirely capable of controlling the infection because of the emergence of multiple escape mutants [11]. Viral mutants differ not only in their infectivity but also in their intracellular pathogenesis. Several lines of evidence showed that the viral core protein plays a key role in development of hepatic steatosis [12], fibrosis [13] and hepatocellular carcinoma [14]. Interestingly, patients were reported to have mild or absent hepatic changes even though viral loads are extremely high [15]. These findings suggest that genomic structure of the quasispecies complex and hence expression of viral proteins in each case are more influential factors on the disease morbidity than the more viral loads. When ALT was used as a marker for liver damage among HCV patients its level showed positive correlation with viral load measured by PCR and branched DNA assays [16]. The same correlation was obtained for both ALT and AST with viral load [17]. Contradictory results were reported [18-20] where significant inverse association between HCV-RNA and ALT levels was observed. From those reports it is evident that the available biochemical, molecular and serological tools, although sensitive and specific, cannot allow clear conclusions with respect to HCV induced pathogenesis in patients infected with different genotypes. On the other hand for a developing country like Egypt where genotype 4 is prevailing [21], there is an increasing need for a supplemental assay besides the costly molecular quantification of viral RNA load for sensitive evaluation of the severity of HCV disease before treatment or during post therapeutic follow ups. This would help better assessment of early response to the expensive peg-interferon/ribavirin combined therapy. The aim of the present work was to evaluate the performance of home made polyclonal mono-specific antibodies raised in rabbits against synthetic peptides of conserved core and envelope sequences as quantitative assays for HCV core and envelope antigens. Measurements of circulating viral structural proteins were tested as markers for the degree of liver damage using ALT and AST levels as indicators. The long term objective was to enhance the cost effectiveness of therapy via better monitoring of patients' response.

## Materials and methods

### 1. Human sera

A total of 65 HCV patients were recruited from the Medical Service Unit of the National Research Center. Diagnosis of those patients was based on, clinical examination, liver enzymes, detectable anti-HCV antibodies in their sera (Axium HCV Rapid test, Florida, U.S.A.), semi quantitative RT-PCR and viral load by bDNA quantitation. A total of 20 sera collected from healthy human subjects having no history of any liver complications, undetectable anti HCV antibodies and negative HCV RNA by RT-PCR in their sera were included as negative controls.

### 2. Methods

#### a. Genotyping

All infected sera included in the study were subjected to genotyping using Versant HCV Genotype Assay (Lipa; Bayer, Germany). Briefly, HCV RNA was extracted, followed by cDNA synthesis using biotinylated random primers specific for the 5' UTR of HCV. The generated biotinylated amplicons were hybridized to immobilized oligonucleotide probes specific for the 5' UTR of different HCV genotypes that are bound to nitrocellulose strips by a poly (T) tail. After hybridization, unhybridized DNA was washed out from the strips that were then treated with alkaline phosphatase labeled streptavidin (conjugate) which bounds then to the biotinylated hybrid. The chromogenic substrate (BCIP/NBT) that allows the formation of a purple/brown precipitate upon degradation by alkaline phosphatase of the conjugate was used for visualization of the banding pattern on the strip.

#### b. Reverse Transcription- Polymerase Chain Reaction (RT-PCR)

Total RNA was extracted from different sera using the acid guanidinium-phenol-chloroform method [[22,23] and [24]] then subjected for RT-PCR [25]. In summary, retrotranscription was performed in 25 μl reaction mixture containing ~400 ng of the extracted RNA, a final concentration of 0.2 mM from each dNTP (MBI Fermentas; St-Leon Rot, Germany), 50 pmol of primers 1CH or 2CH for plus or minus strands respectively and the mixture was preheated at 92°C for 30 sec then chilled on ice. The sequence of the primers used was as follows: 1CH : 5^'^-GTGCACGGTCTACGAGACCTC-3' 2CH: 5^' ^AACTACTGTCTTCACGCAGAA-3'A total of 40U RNasin (MBI Fermentas; St-Leon Rot, Germany).), 20U of Avian Myeloblastosis Virus reverse transcriptase and 2.5 μl 10X enzyme buffer (MBI Fermentas; St-Leon Rot, Germany)) were added to the mixture, the reaction was incubated in the thermal cycler (T1; Whatman Biometra, Goettingen, Germany) at 42°C for 60 min, then the enzyme was inactivated and double stranded cDNA was denatured at 95°C for 10 min and chilled on ice to keep the DNA strands apart. The amplification of the highly conserved 5^'^-UTR sequences was done in 2 rounds of PCR using 2 different pairs of nested primers, one pair in each round. The sequence of the primer pair used in the first round was:

Forward (2CH): mentioned above.

Reverse (P2): 5^'^-TGCTCATGGTGCACGGTCTA-3'

While the sequence of the primer pair used in the second round was:

Forward (F2): 5^'^-GTGCAGCCTCCAGGACCC-3'

Reverse (1TS): 5^'^-GCGACCCAACACTACTCGGCT-3'

Both reactions were carried out in 50 μl volume and the mixture contained 0.2 mM from each dNTP, 10 μM of each primer, 5 μl (10X) reaction buffer containing 1.5 mM MgCl_2_, 10 μl cDNA and 2U *Taq *DNA polymerase (Finnzyme Oy; Espoo, Finland). Amplification was carried out for 30 cycles each included 1 min denaturation at 94°C, 1 min annealing at 55°C and 1 min extension at 72° and the last cycle was attached to a final extension step for 10 min at 72°C followed by soak at 4°C. Amplification products were analyzed by electrophoresis on 1.5% agarose gel in TBE buffer (0.045 M Tris-borate, 0.001 M EDTA, pH 8.0 [26].

#### c. Quantitation of viral RNA load

The assay was performed according to manufacturer's instructions (Versant HCV RNA 3.0 assay bDNA) based on **Elbeik *et al***., [27]

#### d. Preparation of immune complexes and ELISA assays

Immune complexes (ICs) were precipitated according to **Juranic *et al***., [29] by mixing equal volumes of infected or control sera with freshly prepared borate buffered saline (25 mM sodium borate, 100 mM boric acid, 75 mM NaCl, 5 mM EDTA disodium salt, pH 8.4) containing 7% polyethylene glycol (PEG-6000) followed by overnight incubation at 4°C then centrifugation at 10,000 rpm for 20 min. The precipitate was resuspended in 50 μl phosphate buffered saline (PBS) and stored at -20°C till use. ELISA was based on purified polyclonal rabbit IgG raised against synthetic core and envelop (E1) peptides specifically designed based on conserved amino acid sequences among various viral genotypes Core: IPKARRPEGRTWAQPGY, E1: GHRMAWDMM [30]. Those peptides were previously reported by our group to have more than 90 % sensitivity and specificity for detecting HCV IgG antibodies in human sera from infected patients [30]. ELISA was performed according to **Maghraby and Bahgat **[31] with minor modifications to early protocol of **Engvall and Perlman **[32]. The assay was carried out in round bottom polyvinyl microtiter plates (ALTO, Italy). Briefly, plates were coated overnight at 37°C with 100 μl per well of the diluted ICs in coating buffer (1:100; 1 M Na_2_CO_3_; 1 M NaHCO_3_, pH 9.6), washed 3 times with PBS containing 0.05% Tween 20 (PBS-T) then blocked with 100 μl/well of PBS-T containing 1% BSA (PBS-T-BSA) for 2 h at 37°C. After 3 washes with PBS-T, 100 μl of diluted (1:500) rabbit anti-E1 or anti core IgG in PBS-0.05% T-5% -FCS were incubated for 2 h at 37°C. After 3 washes, antibody binding was detected using 100 μl/well from 1:500 diluted peroxidase-labeled anti-rabbit IgG (Sigma Deisenhofen, Germany). For visualization of the immune reaction, 100 μl/well of OPD substrate (Sigma, Deisenhofen, Germany) diluted in substrate buffer (0.1 M citric acid anhydrous; 0.2 M dibasic sodium phosphate, pH 5.0) containing 0.06% H_2_O_2 _were used and plates were left for 10 min at room temperature till color development. The enzymatic reaction was stopped using 40 μl/well 2 M HCl and the change in optical density (OD) was recorded at λ_max _490 nm using a multi-well plate reader (TECAN; SUNRISE, Austria, GmbH).

#### e. Detection of HCV core and E1 by Western blotting

Serum samples of infected and non infected individuals with HCV were subjected to sodium dodecylsulphate polyacrylamide gel electrophoresis (SDS-PAGE; [33] through 4% stacking and 16% resolving gels in 0.75 mm-thick vertical slab gels. Serum samples were diluted, 1: 25 in PBS, mixed with the sample buffer (0.125 M Tris base, 4% SDS, 20% glycerol, 10% mercaptoethanol, and 0.1% bromophenol blue as a tracking dye) and immediately boiled for three minutes. A mixture of reference proteins was run in parallel. Gels were then stained with Coomassie blue. Western blotting was performed as follows: resolved samples separated by SDS PAGE were electro-transferred onto nitrocellulose membranes (0.45 mm pore size). Blotting was carried out at a constant voltage of 60 volts for two hours. The immunoassay was then performed using rabbit anti-HCV core antibodies diluted 1: 100 in blocking buffer with constant shaking. The blots were then washed, followed by incubation for 2 h with goat anti-rabbit IgG alkaline phosphatase conjugate (Zymed) diluted 1:1000 in Tris-buffered saline [TBS; pH 7.4]. The nitrocellulose membrane was then soaked in premixed alkaline phosphatase substrate (5-bromo-4-chloro-3-indolyl phosphate [BCIP], nitro blue tetrazolium [NBT], and 0.1 M Tris buffer, pH 9.6. The color was observed within 10 minutes and the reaction was stopped by the addition of distilled water.

### Statistical analysis

Standard methods were used to calculate sensitivity, specificity, efficiency, and positive and negative predictive values. All parameters were transferred to an IBM PC-AT-compatible computer for analysis using statistical analysis program package Instate Software for Science, version 2.3 (Graphpad Software, Inc., San Diego, Calif.). The Mann-Whitney U test was used to compare the means of two distributions. Fisher's exact test was used to compare the differences between two proportions. *P *values (two-tailed test) of less than 0.05 were considered significant.

## Results

### 1. RT-PCR and genotyping

The RT-PCR results on RNA extracted from 65 patient's sera and 20 uninfected subjects (controls) revealed the presence of HCV RNA in all infected sera whereas none of the controls had any detectable HCV RNA. All patients^,^sera used were confirmed to be genotype 4 (results not shown).

### 2. HCV core and E1 OD levels in the precipitated ICs from HCV patients' sera

**Figure 1:**
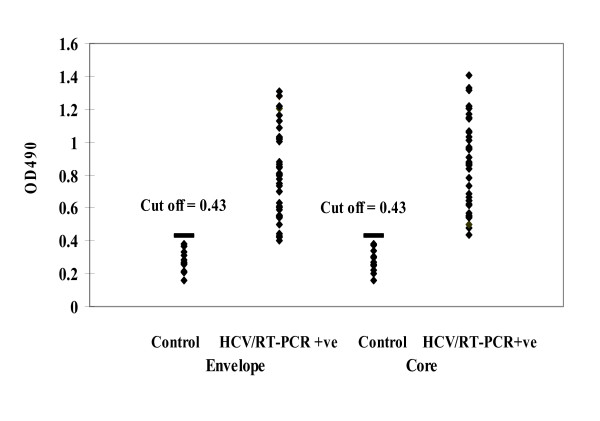
Scattered plot of the OD values corresponding to circulating core and envelop HCV antigens in precipitated immune complexes from viremic patients in comparison to controls. The cut off values for viral antigens were calculated as the mean OD readings obtained from ICs of controls plus 2 × standard deviations and was 0.4. The mean OD readings of viral core and envelop proteins were significantly higher in viremic patients than controls and the P-values for both antigens were (<0.0001) and (<0.0003) respectively.

Precipitated immune complexes from 20 sera of healthy subjects were included as means of negative controls. The cut off value for both core and E1 was calculated (0.4) as the mean OD readings obtained from the immune complexes of the healthy control sera plus 2 × standard deviations. The mean OD readings of both core and E1 antigens were significantly (P < 0.05) higher among the viremic patients when compared to negative controls (Fig. 1). ELISA assay based on the detection of the target HCV core and E1 antigen in serum was developed for the diagnosis of HCV infection. The mean absorbance values of the serum samples were 0.697 and 0.644 for Core and E1 respectively (range, 0.411 to 1.250; all positive) for 65 HCV-positive subjects and 0.286 (range, 0.135 to 0.377; all negative) for 20 healthy human subjects (table 1).

**Table 1:** Mean OD values of both core and envelope circulating HCV antigens in precipitated immune complexes from HCV infected individuals and uninfected healthy controls

**Mean OD values**			
Core		Envelope	
Infected	Control	Infected	Control
0.697	0.286	0.644	0.286
P < 0.0001		P < 0.0003	
Cut off = 0.4			

### 3. Detection of viral core and E1 in HCV patients by western blotting

**Figure 2:**
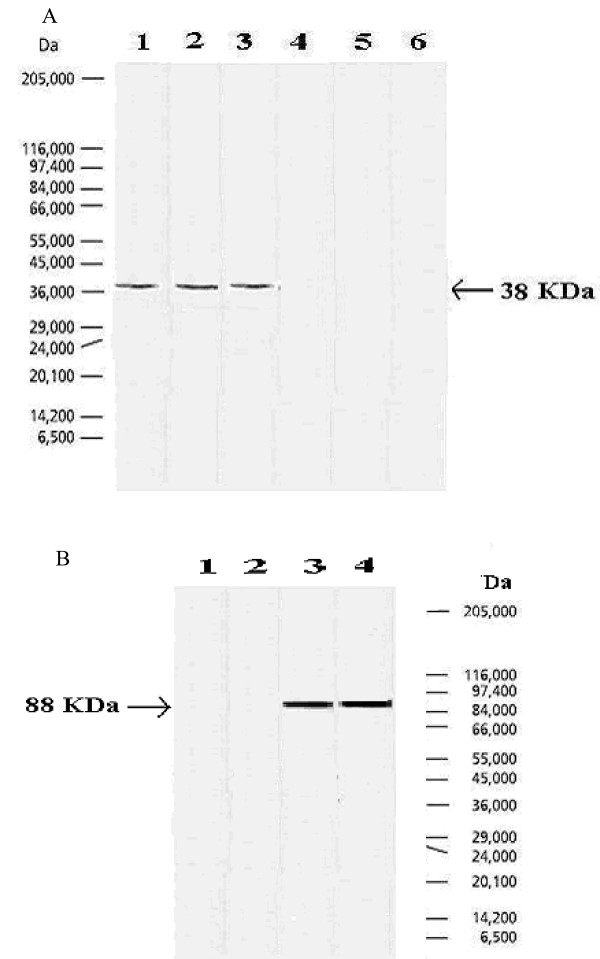
Western blot analysis of serum samples from *Hepatitis C virus *infected and noninfected individuals with antisera monospecific to the Core (Fig. 2a) and E1 (Fig.2b) region of HCV. Fig. 2a. A single band was identified at 38 kDa in serum samples from infected individuals only, lanes 1 to 3, serum samples from three individuals infected with *Hepatitis C virus *; lanes 4 to 6, serum samples from three noninfected healthy individuals. Fig. 2b. A single band was identified at 88 kDa in serum samples from infected individuals only, lanes 3 and 4, serum samples from two individuals infected with *Hepatitis C virus *; lanes 1 and 2, serum samples from two noninfected healthy individuals. Molecular mass bands are not shown but are indicated by arrows.

Sera of HCV infected patients who have been confirmed to be infected with the virus using RT-PCR and had high titer of both core and E1 viral antigens were used for western blot analysis. Sera of healthy individuals were included as negative controls. Results showed that treatment of resolved antigens from infected patients with anti-sera raised against HCV core peptide resulted in visualization of an immunogenic protein band around ~38 kDa (Fig. 2a.) that was undetectable in sera from the healthy controls. On the other hand, treatment of resolved antigens from infected patients with anti-sera raised against HCV E1 peptide resulted in visualization of an immunogenic protein band around ~88 kDa (Fig. 2b).

### 4. Correlation between OD levels of circulating core and E1 antigens in HCV infected subjects

**Figure 3:**
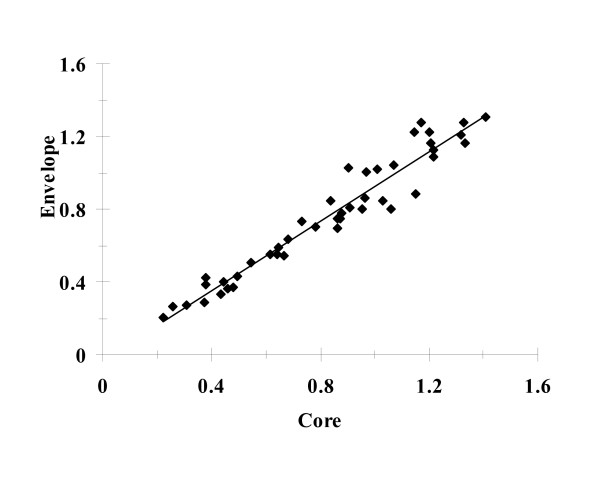
Correlation between levels of core and E1 antigens: ELISA was performed on circulating antigens in 65 HCV infected patients using rabbit anti core or anti E1 as primary antibodies. The OD readings obtained for both antigens were correlated. The results revealed strong positive correlation between levels of both antigens. Correlation coefficient (r = 0.98) and P-value (<0.0001).

OD readings were obtained from ELISA assays on both core and E1 antigens in the sera of 65 patients. Linear regression analysis (Fig. 3) showed a strong positive correlation between OD values of both viral antigens (r = 0.98; P < 0.0).

### 5. Correlation between levels of both HCV core and E1 antigens with viral copy number in HCV infected subjects

**Figure 4:**
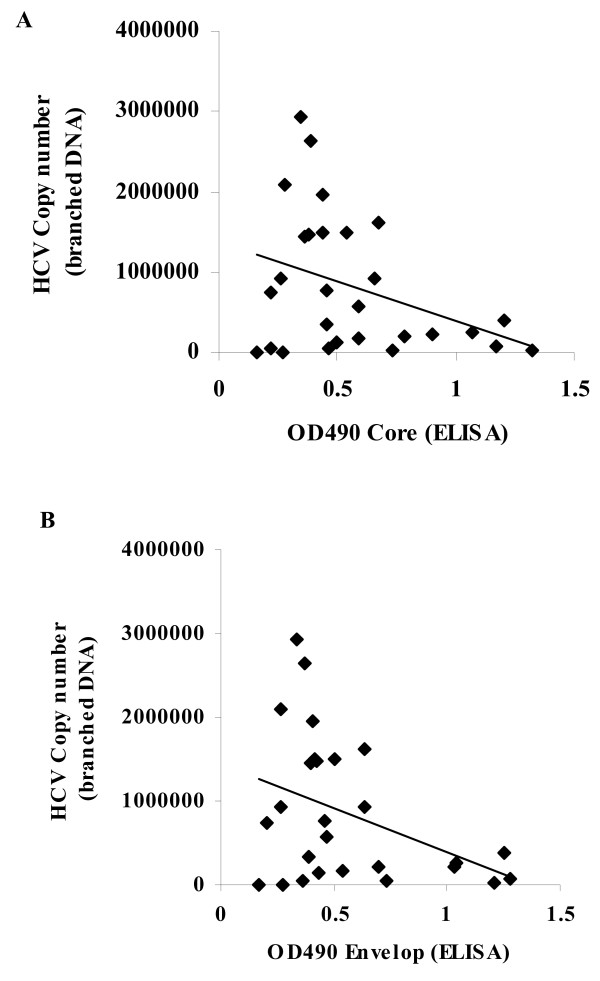
Correlation between levels of both HCV core (A) and envelop (B) antigens with viral copy number quantitation results obtained by branched DNA assay. The results reflected the presence of negative correlation for OD values of both antigens when compared to the branched DNA viral copy number values and the correlation coefficients were (r = -0.36 (A), -0.39 (B)) for both core-branched DNA and envelop-branched DNA. These values were not extremely significant in case of core-branched DNA (P < 0.06) but was clearly significant for envelop-branched DNA (P < 0.05).

When the OD readings corresponding to the reactivities of circulating core (A) and E1 (B) antigens were compared with the viral load as measured by bDNA assay, the results reflected absence of correlation between core antigen and viral-RNA copy number (r = -0.36, P < 0.06) (Fig 4a). Such absence of correlation was also observed between E1 antigen and viral copy number (r = -0.39, P < 0.05) (Fig 4b)

### 6. Correlation between HCV core and E1 antigens with the levels of ALT and AST

**Figure 5:**
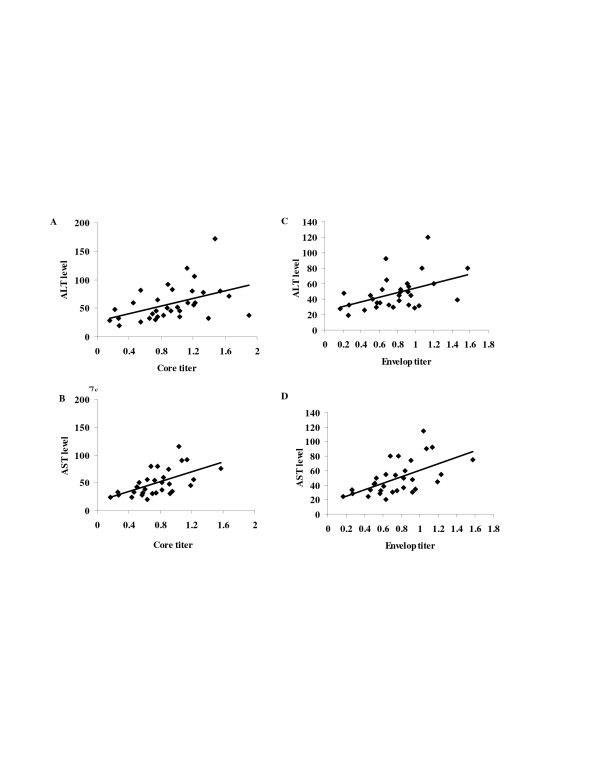
Correlation between levels of both HCV core and E1 antigens with the serum levels of ALT and AST. Results showed generally positive correlation between the level of the core antigen and both enzymes and the correlation coefficient values were (A; r = 0.44, P value is 0.008) for ALT and (B; r = 0.47, P value is 0.003) for AST. And also positive correlation between E1 and both enzymes. The correlation coefficient values were (C; r = 0.43, P is 0.0075) for ALT and (D; r = 0.64, P is 0.0001) for AST.

Comparing the OD readings corresponding to the reactivity of HCV-core antigen with ALT and AST 'levels in patients sera revealed positive correlation (Fig. 5A &5B) between the level of the core antigen and both enzymes (r = 0.44, 0.47 for ALT and AST respectively; P < 0.05 for both). When the OD readings of the E1 levels in patients sera were compared with ALT and AST levels, results showed positive correlation (r = 0.43, 0.64 for ALT and AST respectively; P < 0.05 for both (Fig. 5C &5D).

### 7. Correlation between HCV load with levels of ALT and AST

**Figure 6:**
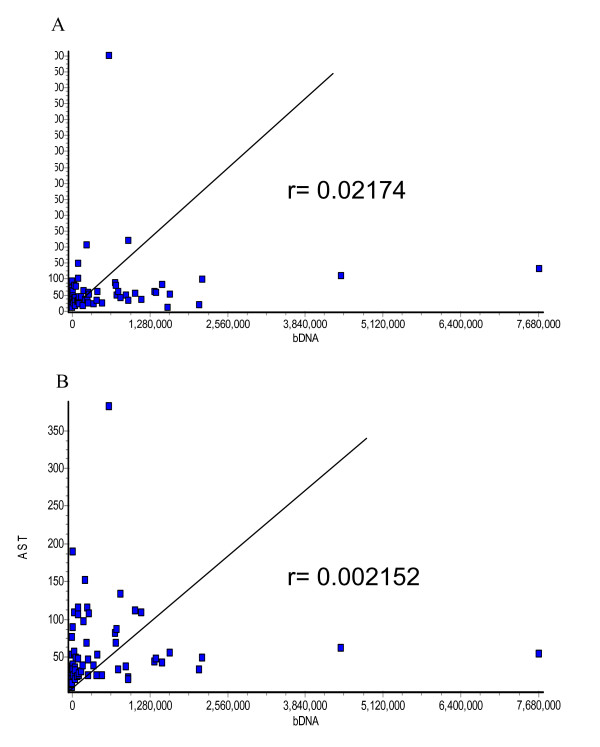
Correlation of HCV load with ALT and AST levels. Linear regression analysis of HCV load with either serum ALT or AST levels was performed in infected patients. Values of correlation coeffecient showed extremely poor correlation between viral copy number and levels of either liver enzyme (A; r = 0.22 for ALT and B; r = 0.002 for AST)

Comparing the HCV load in infected patients with serum ALT and AST levels revealed extremely poor correlation (r = 0.022 and 0.002 respectively) (Fig 6a &6b).

## Discussion

Monitoring the pathogenesis of human HCV disease has been a conflicting issue. Several investigators have looked for laboratory tools that can monitor progress in liver pathogenesis and response to antiviral treatments, yet, the obtained results did not allow clear conclusions and dramatically varied among different HCV genotypes [34,35]. These inter-individual variations in diagnosing active infection, responsiveness to treatment and development of symptoms could be due to the extensive mutational rates in HCV genome followed by emergence of several quasispecies in a single patient [[36-38] and [8]]. Thus the present study was designed to demonstrate the complexity in diagnosing HCV and to discuss whether testing the levels of viral antigens correlates with the severity of the disease and whether the available tools including the quantification of viral load are reliable. Measurements of anti-HCV core and E1 antibodies were expected to be more useful than viral load in better diagnosis of active HCV infection since they will detect viral proteins which reflect the ability of assembling viral particles and subsequent intracellular inflammatory reactions, not only the existence of viral RNA. The anti-synthetic peptide antibodies used in the present study have recently been shown sensitive to detect viral core and E1 antigens in PBMCs derived from HCV infected patients [39]. Furthermore, the conserved viral core and E1 peptides that were used to develop the polyclonal antibodies in rabbits were recognized by human anti- HCV IgG antibodies in more than 90 % of infected patients [30] thus suggesting that these two peptides are immunogenic in humans and rabbits. The results presented herein clearly demonstrate that our polyclonal antibodies are 100% sensitive since they were able to detect viral antigens in all HCV RNA positive cases. Viral genotyping revealed that all cases included contain HCV genotype 4, probably explaining disagreement with previous reports on genotypes 1 and 2 that displayed positive correlation between core levels and viral loads [[40,41] and [42]]. This disagreement could likely be due to differences in the nature and antigenicity of the detected core epitopes in our study than in previous reports. Noteworthy, our anti E1 antibodies were raised specifically against conserved peptides [30] among nearly all the reported viral genotypes. The observed high degree of correlation between core and E1 levels (r = 0.98) supports the earlier hypothesis that both proteins are cleaved simultaneously during processing of viral polyprotein precursor [43]. In addition, since bDNA is an assay testing genomic copy number based on untranslated sequence of the virus, thus from our particular point of view it has nothing to do with expressed viral proteins (whether core or E1), and hence, one can very much accept absence of correlation between circulating viral antigens and viral loads. Also, the over sensitivity of the bDNA can come at the expense of its specificity and may question its value as a proper and realistic tool for monitoring the existence of the virus especially in following up the response to treatment Our hypotheses are not only speculative but were also based on our frequent observations among HCV genotype 4 infected patients with high copy number who neither had pathogenic symptoms, abnormal ultrasound findings nor abnormal profile of liver enzymes (our unpublished data). Noteworthy, immune complexes precipitated from sera of those subjects also showed undetectable levels of HCV core and E1 antigens. Our data clearly demonstrate the absence of correlation between viral copy number as measured by bDNA with either levels of ALT or AST and this was in agreement with **McCormick *et al***., [18]; **Yeo *et al***., [20]; **Anand and Velez**, [44] and contradictory to results obtained by **Beld *et al***., [45]; **Sabin *et al***., [17] and **Hassan *et al***., [16]. Although it seems conflicting that results of **Hassan *et al***., [16] and ours are contradicting while the study populations in both reports were Egyptians, this is not surprising particularly under the random available treatments in Egypt (natural products, liver supportive drugs, antiviral drugs and interferon) which may lead to lowering liver enzymes while having no effect on different viral quasispecies that may exist in infected patients or may result in emergence of new quasispecies with various degrees of aggression. To avoid such conflicts, one has to recruit properly characterized patients who have been certainly receiving no treatment before getting enrolled into such studies. A major scientific advantage of this study is the development of a serological system that can be automated for high throughput analysis. Once this system is available, one can pick up early infected subjects via massive screening programs in heavily infected areas.

HCV antigens were detected in serum, blood mononuclear cells and liver tissues from infected patients [[46,40] and [47]]. In the present study, sera of HCV infected patients were used to provide direct proof on the specificity of the antibodies used in the current study by showing single and discrete bands in western blotting analysis. The immunogenic protein bands around ~38 kDa for core and ~88 kDa for E1 were only found in sera of HCV patients but not in sera from the healthy controls. **Zhang *et al***., [48] found a fusion protein of 32 kDa of HCV core protein exhibiting specific reaction with HCV-positive serum. Also, **Choukhi *et al***., [49] found a 90 kDa band of E1/E2 heterodimer in infected but not in healthy subjects. Further investigations on the structure of the target HCV core and E1 *serum *antigens are required. In conclusion we present a new combination of serological tools correlating with liver enzyme levels that could be utilized as supplemental tests to viral load testing. This new combination of tests would probably allow clinicians to get to better conclusions about not only the severity of liver pathogenesis in viremic patients but also for following up longitudinally the responsiveness of those patients to whichever antiviral therapy by tracing the titers of core or E1 together with viral loads and liver enzyme levels. Such combination of tools should provide significant data on the selection criteria for the initiation of antiviral therapy and also for deciding whether to continue or terminate the selected antiviral treatment. Furthermore, the development of direct immunoassay test for viral activity should allow cheap and efficient screening for acute and early infections in areas where the viral burden is high.

## References

[B1] Rice CM, Fields BN, Knippe DM, Howley PM (1996). Fields Virology.

[B2] Houghton M, Fields BN, Knippe DM, Howley PM (1996). Fields Virology.

[B3] Oldstone MB (1989). Viral persistence. Cell.

[B4] De la Torre JC, Oldstone MB (1996). Anatomy of viral persistence. mechanisms of persistence and associated disease. Adv Virus Res.

[B5] Domingo E, Menendez-Arias L, Quinones-Mateu ME, Holguin A, Gutierrez-Rivas M, Martinez MA, Quer J, Novella IS, Holland JJ (1997). Viral quasispecies and the problem of vaccine-escape and drug-resistant mutants. Prog Drug Res.

[B6] Eigen M (1996). On the nature of virus quasispecies. Trends Microbiol.

[B7] Martell M, Esteban JI, Quer J, Genesca J, Weiner A, Esteban R, Guardia J, Gomez J (1992). Hepatitis C virus (HCV) circulates as a population of different but closely related genomes: quasispecies nature of HCV genome distribution. J Virol.

[B8] Bukh J, Miller RH, Purcell RH (1995). Genetic heterogeneity of hepatitis C virus: quasispecies and genotypes. Semin Liver Dis.

[B9] Hong Z, Beaudet-Miller M, Lanford RE, Guerra B, Wright-Minogue J, Skelton A, Baroudy BM, Reyes GR, Lau JY (1999). Generation of transmissible hepatitis C virions from a molecular clone in chimpanzees. Virology.

[B10] Farci P, Shimoda A, Wong D, Cabezon T, De Gioannis D, Strazzera A, Shimizu Y, Shapiro M, Alter HJ, Purcell RH (1996). Prevention of hepatitis C virus infection in chimpanzees by hyperimmune serum against the hypervariable region 1 of the envelope 2 protein. Proc Natl Acad Sci USA.

[B11] Farci P, Bukh J, Purcell RH (1997). The quasispecies of hepatitis C virus and the host immune response. Springer Semin Immunopathol.

[B12] Koike K, Moriya K (2005). Metabolic aspects of hepatitis C viral infection: steatohepatitis resembling but distinct from NASH. J Gastroenterol.

[B13] Shin JY, Hur W, Wang JS, Jang JW, Kim CW, Bae SH, Jang SK, Yang SH, Sung YC, Kwon OJ, Yoon SK (2005). HCV core protein promotes liver fibrogenesis via up-regulation of CTGF with TGF-beta1. Exp Mol Med.

[B14] Xue KX (2005). Molecular genetic and epigenetic mechanisms of hepatocarcinogenesis. Ai Zheng.

[B15] Moatter T, Hussainy AS, Hamid S, Ahmad Z, Siddiqui S (2002). Comparative analysis of viral titers and histologic features of Pakistani patients infected with hepatitis C virus type 3. Int J Infect Dis.

[B16] Hassan MI, Kassim SK, Ali HS, Sayed el-D A, Khalifa A (2002). Evaluation of nitric oxide (NO) levels in hepatitis C virus (HCV) infection: relationship to schistosomiasis and liver cirrhosis among Egyptian patients. Dis Markers.

[B17] Sabin CA, Emery V, Devereux HL, Griffioen A, Bishop J, Dusheiko G, Yee TT, Herrero-Martinez E, Lee CA (2002). Long-term patterns of hepatitis C virus RNA concentrations in a cohort of HIV seronegative men with bleeding disorders. J Med Virol.

[B18] McCormick SE, Goodman ZD, Maydonovitch CL, Sjogren MH (1996). Evaluation of liver histology, ALT elevation, and HCV RNA titer in patients with chronic hepatitis C. Am J Gastroenterol.

[B19] Trabaud MA, Bailly F, Si-Ahmed SN, Chevallier P, Sepetjan M, Colucci G, Trepo C (1997). Comparison of HCV RNA assays for the detection and quantification of hepatitis C virus RNA levels in serum of patients with chronic hepatitis C treated with interferon. J Med Virol.

[B20] Yeo AE, Ghany M, Conry-Cantilena C, Melpolder JC, Kleiner DE, Shih JW, Hoofnagle JH, Alter HJ (2001). Stability of HCV-RNA level and its lack of correlation with disease severity in asymptomatic chronic hepatitis C virus carriers. J Viral Hepat.

[B21] Tanaka Y, Agha S, Saudy N, Kurbanov F, Orito E, Kato T, Abo-Zeid M, Khalaf M, Miyakawa Y, Mizokami M (2004). Exponential spread of hepatitis C virus genotype 4a in Egypt. J Mol Evol.

[B22] Chomczynski P, Sacchi N (1987). Single-step method of RNA isolation by acid guanidinium thiocyanate-phenol-chloroform extraction. Anal Biochem.

[B23] Fong TL, Shindo M, Feinstone SM, Hoofnaglen JH, Di Bisceglie AM (1991). Detection of replicative intermediates of hepatitis C viral RNA in liver and serum of patients with chronic hepatitis C. J Clin Invest.

[B24] Goergen B, Jakobs S, Symmons P, Hornes E, Meyer zum Buschenfelde KH, Gerken G (1994). Quantitation of HCV-replication using one-step competitive reverse transcription-polymerase chain reaction and a solid phase, colorimetric detection method. J Hepatol.

[B25] El-Awady MK, Abdel Rahman MM, Ismail SM, Amr KS, Omran M, Mohamed SA (2003). Prediction of relapse after interferon therapy in hepatitis C virus-infected patients by the use of triple assay. J Gastroenterol Hepatol.

[B26] Maniatis T, Fritsch EF, Sambrook J (1982). Molecular Cloning: A laboratory Manual. Cold Spring Harbor Laboratory, Cold Spring Harbor, New York.

[B27] Elbeik T, Markowitz N, Nassos P, Kumar U, Beringer S, Haller B, Ng V (2004). Simultaneous runs of the Bayer VERSANT HIV-1 version 3.0 and HCV bDNA version 3.0 quantitative assays on the system 340 platform provide reliable quantitation and improved work flow. J Clin Microbiol.

[B28] Lock RJ, Unsworth DJ (2000). Measurement of immune complexes is not useful in routine clinical practice. Ann Clin Biochem.

[B29] Juranic Z, Babovic N, Tomasevic Z (1997). Levels of immune complexes in fresh and frozen serum and plasma detected by precipitation with polyethylene glycol in patients with malignant diseases. Srp Arh Celok Lek.

[B30] El Awady MK, El-Demellawy MA, Khalil SB, Galal D, Goueli SA (2002). Synthetic peptide-based immunoassay as a supplemental test for HCV infection. Clin Chim Acta.

[B31] Maghraby A, Bahgat M (2004). Immunostimulatory Effect of Coumarin Derivatives Before and After Infection of mice with the Parasite *Schistosoma mansoni *: *In Vivo Study*. Drug Research "Arzneimittelforschung".

[B32] Engvall E, Perlman P (1971). Enzyme-linked immunosorbent assay (ELISA). Quantitative assay of immunoglobulin G. Immunochemistry.

[B33] Laemmli UK (1970). Cleavage of structural proteins during the assembly of the head of bacteriophage T4. Nature.

[B34] Hnatyszyn HJ (2005). Chronic hepatitis C and genotyping: the clinical significance of determining HCV genotypes. Antivir Ther.

[B35] Pawlotsky JM (2005). Current and future concepts in hepatitis C therapy. Semin Liver Dis.

[B36] Hijikata M, Kato N, Ootsuyama Y, Nakagawa M, Ohkoshi S, Shimotohno K Hypervariable regions in the putative glycoprotein of hepatitis C virus. Biochem Biophys Res Commun.

[B37] Weiner AJ, Brauer MJ, Rosenblatt J, Richman KH, Tung J, Crawford K, Bonino F, Saracco G, Choo QL, Houghton M (1991). Variable and hypervariable domains are found in the regions of HCV corresponding to the flavivirus envelope and NS1 proteins and the pestivirus envelope glycoproteins. Virology.

[B38] Martell M, Gomez J, Esteban JI, Sauleda S, Quer J, Cabot B, Esteban R, Guardia J (1999). High-throughput real-time reverse transcription-PCR quantitation of hepatitis C virus RNA. J Clin Microbiol.

[B39] El-Awady MK, Tabll A, EL-Rashdy MR, Youssef S, Omran MH, Thakeb F, EL-Demellawy M (2005). Flow Cytometric detection of hepatitis C virus antigens in infected peripheral blood leucocytes: binding and entry. World J Gastroenterol.

[B40] Orito E, Mizokami M, Tanaka T, Lau JY, Suzuki K, Yamauchi M, Ohta Y, Hasegawa A, Tanaka S, Kohara M (1996). Quantification of serum hepatitis C virus core protein level in patients chronically infected with different hepatitis C virus genotypes. Gut.

[B41] Dickson RC, Mizokami M, Orito E, Qian KP, Lau JY (1999). Quantification of serum HCV core antigen by a fluorescent enzyme immunoassay in liver transplant recipients with recurrent hepatitis C. Clinical and virologic implications. Transplantation.

[B42] Maynard M, Pradat P, Berthillon P, Picchio G, Voirin N, Martinot M, Marcellin P, Trepo C (2003). Clinical relevance of total HCV core antigen testing for hepatitis C monitoring and for predicting patients' response to therapy. J Viral Hepat.

[B43] McLauchlan J, Lemberg M, Hope G, Martoglio B (2002). Intramembrane proteolysis promotes trafficking of hepatitis C virus core protein to lipid droplets. The EMBO journal.

[B44] Anand BS, Velez M (2004). Assessment of correlation between serum titers of hepatitis c virus and severity of liver disease. World J Gastroenterol.

[B45] Beld M, Penning M, McMorrow M, Gorgels J, van den Hoek A, Goudsmit J (1998). Different hepatitis C virus (HCV) RNA load profiles following seroconversion among injecting drug users without correlation with HCV genotype and serum alanine aminotransferase levels. J Clin Microbiol.

[B46] Attallah AM, Ismail H, Tabll AA, Shiha GE, El-Dosoky I (2003). A novel antigen detection immunoassay for field diagnosis of hepatitis C virus infection. J Immunoassay Immunochem.

[B47] Shiha GE, Zalata KR, Abdalla AF, Mohamed MK (2005). Immunohistochemical identification of HCV target antigen in paraffin-embedded liver tissue: reproducibility and staining patterns. Liver Int.

[B48] Zhang YM, Li M, Dong WQ, Wu YS, Tian ZW, Wu XB (2003). Cloning and expression of hepatitis C core protein gene. Di Yi Jun Yi Da Xue Xue Bao.

[B49] Choukhi A, Pillez A, Drobecq H, Sergheraert C, Wychowski C, Dubuisson J (1999). Characterization of aggregates of hepatitis C virus glycoproteins. J Gen Virol.

